# Structure and Function of a Provincial Renal Pharmacy Program: Applying the Chronic Care Model to Address Equitable Access to Medication and Pharmacy Services

**DOI:** 10.1177/20543581231177840

**Published:** 2023-06-07

**Authors:** Elnaz Roohi, Clifford Lo, Dan Martinusen, Adeera Levin

**Affiliations:** 1 Department of Experimental Medicine, Faculty of Medicine, The University of British Columbia, Vancouver, Canada; 2 BC Renal Agency, Vancouver, Canada; 3 Faculty of Pharmaceutical Sciences, The University of British Columbia, Vancouver, Canada; 4 Royal Jubilee Hospital, Island Health, Victoria, Canada; 5 Division of Nephrology, The University of British Columbia, Vancouver, Canada

**Keywords:** Health services research, provincial pharmacy services program, provincial health services authority, chronic kidney disease (CKD), chronic care management model

## Abstract

**Purpose:**

We described the rationale, structure, design, and components of a provincial pharmacy services network for patients with kidney disease as a model for enabling equitable access and universal care to pharmacy services and medications across a wide range of clinical conditions, and geographic expanse in British Columbia (BC).

**Sources of Information:**

These include minutes from 53 Pharmacy Services and Formulary (PS&F) Committee meetings held from 1999 to November 2022, documentation available on the British Columbia Renal (BCR) website, direct observation and participation in committee meetings, as well as interviews with key individuals involved in different aspects of the program.

**Methods:**

We reviewed documents and data describing the evolution, rationale, and functioning of the BCR provincial pharmacy services system and used a variety of sources as mentioned above. In addition, a qualitative thematic synthesis of reports of chronic care models (CCMs) was conducted to map the program components into the chronic disease management models.

**Key Findings:**

The components of the provincial pharmacy program (PPP) include (1) a PS&F committee, with interdisciplinary and geographical representation; (2) a community of dispensing pharmacies with standardized protocols and information; (3) a dedicated medication and pharmacy services budget, and regular evaluation of budget, outcomes, and performance; (4) provincial contracts for specific medications; (5) communication and education; and (6) information management system. Program components are described in the context of chronic disease management models. The PPP includes dedicated formularies for people with kidney disease at different points in the disease trajectory, including those on and off dialysis. Equitable access to medications is supported across the province. All medications and counseling services are provided to all patients registered in the program, through a robust distributed model, including community- and hospital-based pharmacies. Provincial contracts managed centrally ensure best economic value, and centralized education and accountability structures ensure sustainability.

**Limitations:**

Limitations of the current report include lack of formal evaluation of the program on patient outcomes, but this is relative as the intention of this article is to describe the program which has existed for over 20 years and is fully functional. Formal evaluation of a complex system would include by costs, cost avoidance, provider, and patients’ satisfaction. We are developing a formal plan for this reason.

**Implications:**

The PPP is embedded in the provincial infrastructure of BCR and enables the provision of essential medications and pharmacy services for patients with kidney disease throughout the spectrum. The leveraging of local and provincial resources, knowledge, and expertise to implement a comprehensive PPP, ensures transparency and accountability and may serve as a model for other jurisdictions.

## Abrégé

**Contexte:**

Nous avons décrit la raison d’être, la structure, la conception et les composantes d’un réseau provincial de services en pharmacie pour les patients atteints d’insuffisance rénale comme un modèle permettant des soins universels et un accès équitable aux services pharmaceutiques et aux médicaments dans un large éventail de conditions cliniques et d’étendues géographiques en Colombie-Britannique (C.-B.).

**Sources:**

Les procès-verbaux de 53 réunions du Pharmacy Services and Formulary Committee tenues entre 1999 et novembre 2022, la documentation disponible sur le site BC Renal, l’observation directe et la participation aux réunions du comité, ainsi que les entretiens avec des personnes clés impliquées dans différents aspects du program.

**Méthodologie:**

Nous avons examiné les documents et les données décrivant l’évolution, la raison d’être et le fonctionnement du système provincial de services pharmaceutiques BC Renal et nous avons utilisé diverses sources, comme mentionné ci-dessus. Une synthèse thématique qualitative des rapports sur les modèles de soins chroniques (MSC) a également été réalisée afin d’intégrer les composantes du program aux modèles de gestion des maladies chroniques.

**Principaux résultats:**

Les composantes du program provincial de pharmacie (PPP) comprennent: 1) un comité des services pharmaceutiques et des listes de médicaments, avec une représentation interdisciplinaire et géographique; 2) une communauté de pharmacies d’officine disposant de protocoles et de renseignements normalisés; 3) un budget dédié aux médicaments et aux services pharmaceutiques, ainsi qu’une évaluation régulière du budget, des résultats et du rendement; 4) des contrats provinciaux pour certains médicaments particuliers; 5) des structures de communication et d’éducation; et 6) un système de gestion de l’information. Les composantes du program sont décrites dans le contexte des modèles de gestion des maladies chroniques. Le PPP comprend des formulaires dédiés pour les personnes atteintes d’insuffisance rénale à différents points de la trajectoire de la maladie, qui sont sous dialyze ou non. Un accès équitable aux médicaments est assuré dans toute la province. Tous les médicaments et les services de conseil sont fournis à tous les patients inscrits au program, par le biais d’un robuste modèle de distribution, comprenant des pharmacies communautaires et hospitalières. Des contrats provinciaux gérés de façon centralisée assurent la meilleure valeur économique, et les structures centralisées d’éducation et de responsabilisation assurent la durabilité.

**Limites:**

L’absence d’évaluation formelle du program sur les résultats des patients, bien que cela soit relatif puisque l’intention de cet article est de décrire un program pleinement fonctionnel qui existe depuis plus de 20 ans. L’évaluation formelle d’un système complexe porterait sur les coûts, les économies de coûts, la satisfaction des prestataires et des patients. Nous sommes en processus d’élaboration d’un plan formel.

**Résultats:**

Le PPP est intégré à l’infrastructure provinciale BC Renal et permet la fourniture de médicaments et de services pharmaceutiques essentiels aux patients atteints de l’ensemble du specter de l’insuffisance rénale. L’exploitation des ressources, des connaissances et de l’expertise locales et provinciales pour mettre en œuvre un PPP complet garantit la transparence et la responsabilisation, et peut servir de modèle à d’autres administrations.

## Introduction

Chronic kidney disease (CKD), with a prevalence of 13.4% (11.7%-15.1%) is a frequently treated condition within health care systems, both universally and within Canada.^1^ Globally, the number of patients with all CKD categories reached approximately 850 million^2^ which is reported to be more than those having diabetes, chronic obstructive pulmonary disease, asthma, osteoarthritis, or even depressive disorders.^3^ Chronic kidney disease is known to have a great impact on the global burden of morbidity and mortality as it directly increases the risk of developing cardiovascular diseases, diabetes, and end-stage kidney disease.^4,5^ According to the Global Burden of Diseases study, CKD resulted in 7.3 million years lived with a disability (YLD)s, 28.5 million years of life lost from mortality (YLL)s, and 35.8 million disability-adjusted life years (DALYs) in 2017.^3^ Medication management for specific conditions and complications of CKD is complex, requires knowledge and access to multiple medications and is fundamental to outcomes of patients with kidney disease. Treatment costs for CKD have been increasing with the emergence of new pharmacotherapies enabling the long-term application of life-saving, though costly therapies for patients with end-stage kidney disease.^6^ The number of people receiving kidney pharmacotherapeutics has exceeded 2.5 million worldwide and is projected to double to 5.4 million by 2030^7^; nevertheless, there is a shortage of kidney care services in many countries and an estimated 2.3 to 7.1 million adults have died prematurely due to limited access to kidney therapies.^7^

Within Canada, and worldwide a variety of organizational structures exist to manage CKD populations, within different health care systems (e.g. Ontario Renal Network (ORN),^8^ Northern and Southern Alberta Renal Program,^9^ British Columbia Renal (BCR)).^10^ Funding and infrastructure for the life support services that these organizations provide are variable across provinces. Canada’s system of prescription drug coverage involves a mix of public and private insurance plans that differ in terms of eligibility, patient charges, and drugs covered (formularies).^11^ Each province offers some form of public subsidy for prescription medications and the public drug plans offered by provinces vary in terms of the patient population that is covered.^12^ British Columbia Renal, previously known as BC Provincial Renal Agency has a unique comprehensive organizational structure and funding model for kidney care in Canada, which includes complete pharmacy services and medications, available at no cost to patients. British Columbia Renal funds, facilitates distribution and manages medications for patients across the spectrum of CKD.

**Figure 1.**
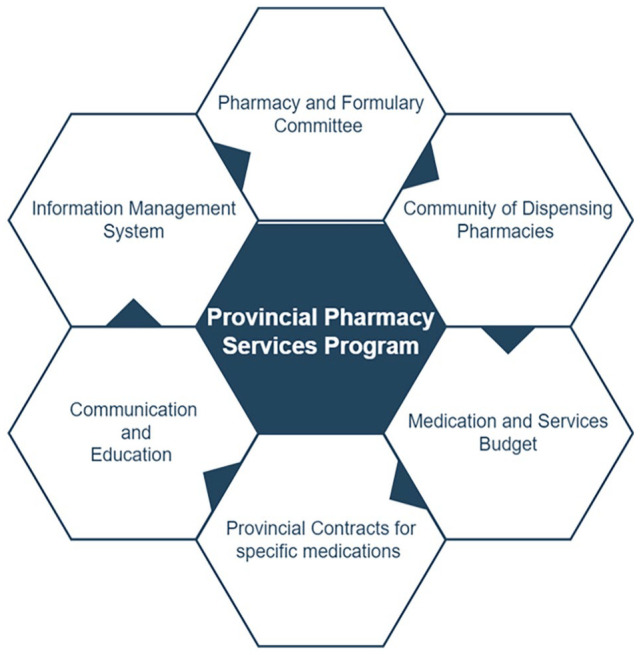
Components of the British Columbia’s pharmacy services program for kidney patients.

In partnership with HA renal programs, BCR manages an annual pharmacy budget of approximately $25 million to accommodate pharmacy services program (medications and infrastructure) for approximately 22,000 patients with kidney disease. Pharmacy services provided by authority-based renal pharmacists are part of a separate operating budget. This is achieved with implementation of an activity-based funding model^12^ that enables flexible and equitable access to care based on each patient’s needs, as well as an accurate assessment of costs. This funding model is focused on cost-effective care, delivered by multidisciplinary teams, and supports province-wide best practices and equitable access to all aspects of kidney care, including medications. Patients are served by pharmacy services programs in specific geographic HAs. British Columbia Renal is a planning and funding body and is responsible for all aspects of direct delivery of pharmacy services care to patients throughout the course of their kidney disease. By creating a network that includes all HA renal programs, as well as a range of provincial committees, BCR supports timely and equitable access to kidney medications and pharmacy services and provide a venue to collaboratively address common challenges that may be faced as well as opportunities to improve patient care. This Provincial Pharmacy Services Program has 6 components (Figure 1):

1. A Pharmacy Services And Formulary (PS&F) Committee, with interdisciplinary and geographical representation.
2. A community of dispensing pharmacies with standardized protocols and information.
3. A dedicated medication and pharmacy services budget, and regular evaluation of budget, outcomes, and performance.
4. Provincial contracts for specific medications.
5. Communication and education.
6. Information management system.

The foundation of these components is based on the elements of the Wagner chronic care model (CCM)^13^ and is in line with BC’s Pharmaceutical Care Management Strategy.^14^ Wagner’s CCM and its components have been previously adopted and evaluated in numerous studies, with results indicating improvement of patients’ care and outcomes together with reduction of care utilization and costs.^15-17^

The aim of this article is to describe the structure and function of this provincial renal pharmacy services program in BC, Canada. We describe here the rationale, design, and the 6 components of a provincial pharmacy program (PPP) for patients with kidney disease as a model of health care that could deliver integrated management of this prevalent chronic disease within the context of pharmacy services. We also map our program components to Wagner’s CCM elements. This article may provide practical guidance for health care program managers, policy-makers, stakeholders, and researchers on how to plan and deliver high-quality services for patients with kidney disease and facilitate adoption in other jurisdictions.

## Methods

All data and documents describing the evolution, rationale, structure, and functioning of the BCR provincial pharmacy services system were reviewed. We used a variety of sources including minutes from 53 PS&F Committee meetings held from 1999 to November 2022, documentation available on the BCR website, direct participation, and observation of the committee meetings, as well as interviews with key individuals involved in different aspects of the program. In addition, a qualitative thematic synthesis of reports of CCMs was conducted to map the program components into the chronic disease management models.

### Rationale

The PPP has been designed to ensure that all patients with kidney disease who require medication to modify disease trajectories, treat symptoms, or address deficiencies or excesses of specific metabolic or hormonal parameters are funded to receive those medications without charge so as to remove cost as a barrier to treatment. Those on dialysis receive complete coverage of all formulary medications related to dialysis, symptoms, and blood pressure management, and those not on dialysis receive medications specifically related to impaired kidney function. British Columbia Renal’s pharmacy services program intends to supplement the robust system existing in BC, which ensures that all get reimbursed for most medications. Most nondialysis CKD patients’ blood pressure and diabetes medication are funded through Fair Pharmacare program, which includes thresholds for deductibles based on income, or through private insurance companies.

In addition, there is coverage of disease-modifying therapies for the treatment of glomerulonephritis (GN) and autosomal dominant polycystic disease (ADPKD). Not covered for any patient are medications to treat other conditions such as diabetes, or thyroid disease (see website for the complete list: http://www.bcrenal.ca/health-professionals/clinical-resources/pharmacy-formulary).

Funding for pharmacy services, in addition to medications, is included in the BCR funding model, equitable across all locations and patient types. Evidence-based care, accountability, equitable access and fiscal responsibility for public dollars are core values of pharmacy services program; which is overseen by the PS&F committee.

### Design

The design of the Provincial Pharmacy Services model follows a hub and spoke model where there are centralized activities (assessment, education, contract management, analytics, and funding) and decentralized activities (patient counseling and medication delivery) so as to ensure efficient and effective management of services to patients, reduce redundancies, and maintain accountability.

## The 6 Components of the BCR’s Pharmacy Program

### PS&F Committee

#### Overall Structure and Function

The PS&F Committee is the committee which oversees the evidence-based formulary, advises on strategy and provides oversight to the whole program, and is not the same as Pharmacy and Therapeutics Committees found in most institutions. Specifically, the structure and scope of the PS&F Committee is intended to facilitate most appropriate use by creating protocols and guidelines and support access to appropriate medications, not just “formulary” review and approval. The PS&F committee has multidisciplinary, geographically diverse membership, including kidney care providers, nephrologists, dieticians (from rural and urban settings) pharmacists (clinical renal pharmacists representing various renal programs and community pharmacists), biostatistician/data management personnel (by invitation), financial lead, and representatives from BCR executive team. Individuals use their expertise, time, and experience to inform protocols, contracts, and implementation issues to BCR committees and working groups. Central to the PS&F committee is the oversight as to medication and oral nutritional supplement coverage and access for kidney patients across the spectrum (nondialysis, GN, and ADPKD). The committee periodically reviews the formulary and updates medication and oral nutritional supplement list to match the current knowledge and evidence to provide access to appropriate medications for patients with kidney disease. Evidence for existing and new pharmacotherapies, and submissions from pharmaceutical companies for new medications (which are already approved by Health Canada) are reviewed to justify funding support by BCR, either directly, or indirectly. The PS&F committee members inform and advise on policies on the use of medications and therapies in keeping with best practices following established principles to optimize patients’ care within available resources, evidence review to identify medications and treatment strategies most medically appropriate and cost-effective and to evaluate the impact of strategies and changes in strategies.

#### Activities of the PS&F committee

*(a*) *Review and update the formulary based on combination of effectiveness, safety, value, and in line with the latest recommendations and best practices.*

The PS&F committee defines a comprehensive and justified list of appropriate medications and nutritional supplements essential for the care of kidney patients. British Columbia has the most comprehensive kidney medication coverage in Canada, with 3 drug formularies that ensure (1) dialysis patients, (2) patients with kidney disease not on dialysis, and (3) patients with specific conditions (e.g. GN and ADPKD) have access to essential disease-modifying medications. The latest BCR formulary for all the mentioned groups of patients can be accessed via the BCR website (BCRenal.ca).^18-21^

Two broad categories of medications are funded by BCR as follows:

1. Chronic medications for chronic conditions due to CKD. British Columbia Renal’s pharmacy services program funds medications treating chronic comorbid conditions that have been developed due to CKD. Supportive care medications for conditions impacting life due to kidney conditions/dialysis are also funded (e.g. antiemetics, laxatives, and anemia treatments).
2. Medications that will alter the decline of kidney function in certain disease states.

Of note, BCR’s pharmacy services program provides access to medications for patients who have an eGFR<15 mL/min and are not yet on dialysis or those who have chosen conservative care. The coverage extends to include medications recommended in the BCR symptom management protocols for all patients with advanced CKD (eGFR < 15 mL/min) regardless of treatment modality chosen. The extension of funding in 2017 for patients who choose conservative care, instead of dialysis, is intended to ensure there is no financial disadvantage to choosing this pathway with regards to affording symptom relief medications. This decision to extend funding for specific medications irrespective of dialysis was arrived at after considering equity and appropriateness.

There are formal and transparent processes used to add or remove drugs to the formulary. Factors considered include strength of evidence, cost-effectiveness, clinician input, patient input, equity, budgetary impact analysis, value added activities, and strategic priorities.

(*b*) *Promote appropriate use of medications specific for kidney patients through identifying best practices, develop and disseminate protocols, and enabling implementation in the province.*

British Columbia’s Renal pharmacy services program strives to facilitate implementation of the most appropriate medication use by the development of innovative protocols, algorithms, and guidelines using best evidence, clinical expertise, existing guidelines, and knowledge of local resources. Thus, a number of complex clinical conditions are managed in standardized manner. Examples include but are not confined to dialysis and nondialysis anemia management protocols,^22^ protocols for Alteplase Administration for Occluded Peritoneal Dialysis Catheter,^23^ and treatment of metabolic bone disease.^24^

British Columbia Renal’s pharmacy services program with the oversight of PS&F committee has implemented several successful initiatives as follows:

##### Medication reconciliation (Med Rec)

Launched in 2009, the medication reconciliation program which was the first of its kind for chronic outpatients in BC was developed to enable complex outpatient management to be conducted safely.^25^ Patients with kidney disease are inherently at a heightened risk of adverse drug reactions (ADRs) and occurrence of medication errors due to polypharmacy, comorbidities,^26^ and altered medication pharmacokinetics^27^ relevant to kidney failure. Thus, the impact of Med Rec on patients’ outcomes is enormous. This initiative has received numerous awards—Health Employers Association of BC (HEABC) Award of Merit (2010), BC Patient Quality & Safety Award (2011), Leading Practice Designation by Accreditation Canada (2011), Canada Health Infoway Trailblazer Award (2012), and the Canadian Association of Hospital Pharmacists (CSHP) National Award (2012).

##### Access to immunosuppressive agents for people with biopsy-proven GN

Within the context of initiatives, the GN network was created to coordinate provincial GN health services delivery including pharmacy services. In 2013, the GN network surveyed BC nephrologists and 86% reported poor access to insurance coverage of immunosuppressant medications, and 98% felt that better immunosuppressant coverage would improve care.^28^ A year later, a formulary for immunosuppressive agents was created. Patients now have access to standardized care protocols, guidelines and funding for immunosuppressive agents via a dedicated GN formulary. Protocols are regularly reviewed and updated, and as new evidence becomes available, new compounds are assessed and integrated.^29^ In 2015, the GN Formulary was awarded a BC Patient Quality & Safety Award in the Returning to Health and Wellness category.^30^

##### Access to diseases-specific or disease-modifying medications for patients with ADPKD

By leveraging internal network structures such as the provincial BC ADPKD Network, standardization of assessment, monitoring, evaluation, and access to the disease-modifying therapy, Tolvaptan, is available.^31^

(c) *Ensure that patients have equitable access to medication.*

All formulary medications are fully funded for all patients registered with BCR. If receiving hemodialysis, the dispensed medications are delivered to dialysis units and if on home-based therapies, medications are delivered to patients’ homes, all at no cost. For those individuals who are not on dialysis, approved medications are supplied from a dedicated vetted community pharmacy, accessible to those patients.

### A Community of Dispensing Pharmacies

#### Rationale

The rationale for having dedicated distribution pharmacies is to address the following issues with the aim of strengthening and transforming delivery systems to provide more effective, efficient, and timely care: (1) certainty of communications, (2) standardization, (3) accountability, and (4) guaranteed education.

#### Design

Contract management services facilitate medication distribution to kidney outpatients in the province. A group of community pharmacies are contracted to provide care to BCR patients, selected based on a formalized process. The Provincial Health Services Authority (PHSA)/BCR invites proposals from community pharmacies with existing infrastructure and within the qualifying area (located near a hemodialysis unit) to provide provincial kidney medications “Dispensing Services” to all patients with kidney disease in BC and that may include distribution of kidney medications to patients’ homes and community and in-center dialysis units across BC. The community pharmacy dispensing services and responsibilities together with BCR responsibilities are outlined in Table 1.

**Table 1.** The Community Pharmacy Dispensing Services & Responsibilities Together With BCR Responsibilities.

Community pharmacy responsibilities	BCR responsibilities
**Ordering of medications specific to the renal formulary**	Timely reimbursement of pharmacies for services rendered
**Providing renal medication service to all patients (i.e. CKD, hemodialysis, peritoneal dialysis)**	Provision of educational support to the pharmacy
**Warehousing, replenish, and management of stock**	Provision of patient medication information pamphlets to the pharmacy
**Patient/clinic receipt of orders and dispensing services**	Provision of ongoing assistance to community pharmacies to improve medication outcomes for BCR patients
**Ordering distribution/shipping to patients’ homes or renal clinics across BC**	Conduct of quality improvement programs
**Distribution to renal dialysis units and home patients**	
**Specific financial information**	
**Counseling and reviewing of drug information as required with the patient, and as per pharmacy best practices**	
**Ensuring a tracking system which permits identification and solution generation to problems with timeliness of distribution**	
**Conducting medication reconciliation as the regional renal program requests**	

*Note.* BCR = British Columbia Renal; CKD = Chronic Kidney Disease.

A component of the contract with community pharmacists includes a commitment to develop expertise in kidney pharmacotherapy, including attending at least a half day training session, and attendance at various educational events including the annual BC Kidney Days (BCKD) professional group session.

Selection of community pharmacies is conducted through a request for proposals (RFPs) process—an open and transparent process, in keeping with best corporate business practices. The process established ensures that community pharmacies are available to provide high-quality and consistent services to the kidney community for the entire contracted period. Thus, patients with kidney disease are able to obtain medications from a BCR-contracted pharmacy of their choice without charge. Medications funded by BCR are supplemental to other existing payers and programs. British Columbia is the only province in Canada that offers such comprehensive pharmacy services to patients with kidney disease, which supports delivery and distribution of reno-pharmacotherapies, and enables evidence-based, best-practice-guided, equitable access for patients with kidney disease across all disease trajectories.

##### Oversight/evaluation of quality of community services

Once contracts are established with the qualified community pharmacies for the supply of the medications or services to patients with kidney disease, there is a process for regular review of deliverables. The review is aimed at ensuring rigorous regular evaluation of key components of the contract: timely access to medications, accurateness of medication delivery, use of BCR-specific education/information materials for patients, compliance rate, and billing accuracy.

Reports, correspondence, documents, and records relating to the performance of the services are reviewed regularly (at least annually) by the PHSA/BCR and are fed back to the pharmacists. Complaints or issues are addressed in real time as identified through centralized mechanisms. These reviews ensure the community pharmacists provide high-quality performance during the contract period. Performance metrics include but are not limited to quoted on-time delivery of medications including fill rates on committed volume contracts, response times, financial impact to support the required delivery dates, contract management responsibilities, problem-solving capabilities, respect for PHSA/BCR policies, ethical conduct and other attributes of leading organizations. We do work with the contracted community pharmacies to ensure the development, provision, and maintenance of a quality assurance plan acceptable to PHSA/BC. Each supplying community pharmacist commits to upholding systemic approach to quality service delivery, and overtly addressing identified deficiencies. These commitments are articulated formally within the contracts. Comprehensive quality assurance reports detailing adherence to the service standards, quality monitoring scores, and quality assurance plan activity are provided to PHSA/BCR on a monthly basis.

*Corrective Actions*: If a concern is raised about a BCR-contracted pharmacy (from patients, health care personnel (HCP) or facility), an investigation is initiated, and corrective action is informally communicated. If further corrective action is required, a formal process is initiated using PHSA Supply Chain to engage with the pharmacy in a formal dispute resolution mechanism (series of 3 demand letters). Beyond that, BCR would work to identify a new pharmacy to initiate a contract with and transfer care over to that new pharmacy.

### A Dedicated Budget for Medication and Pharmacy Services

#### Rationale

To provide equitable access to medication and pharmacy services for patients with kidney disease, regardless of where they live and what level of care they need, a dedicated budget is allocated for the pharmacy services program. Budgeting allows BCR to account for factors such as volume growth assumptions, feedback and input from key sectors, and the ability to adjust at the time when inputs and circumstances change.

The dedicated budget addresses 2 key elements for improving quality of health care and clinical outcomes, which have been identified to be universal coverage and health care that is without charge at the point of service.^32^ In the absence of these 2 elements, patients may delay seeking health care due to costs. Indeed, catastrophic health expenditure is a critical factor in impoverishing patients^32^ and universal coverage and health care access contribute tremendously to reduction of inequities in health care.^33^

#### Design

Within the life support budget dedicated to the care of patients with kidney disease, there is a specific budget for pharmacy and formulary services. This dedicated budget includes payment for medications and dispensing fees, and the support of HA, facility-based pharmacy personnel, as an adjunct to the community pharmacy dispensing system. Funding letters delineating the amount and roles of these pharmacy personnel are shared with each HA.

Medication budgets are based on calculations generated using patient-year data and average medication usage by patient modality. Funding for the pharmacy services is included in the HA transfer payment, while medication payments are managed centrally, as is funding for the infrastructure support for education, communication, and evaluation.

The role of the HA-based renal pharmacists is to ensure on-sight facility-based adherence to best practices, facilitate education of patients, and HCPs, and to provide clinical pharmacy services to patients, supplemental to that received from community pharmacists. Whilst there is an allocation of renal pharmacists per HA, given hiring and personnel challenges, the numbers are variable. Pharmacy services are calculated based on an established activity-based funding model and can be used for pharmacist services, including pharmacy technicians and other trained professionals.

### Provincial Contracts for Specific Medications

#### Rationale

The rationale for having provincial contracts for specific medications includes value for money, ease of management, central distribution, and stability of supply. Provincial contracts (and value add for regional renal program funds) enable BCR to leverage cost savings. Value for money is driven through lower medication costs, achieved through value add contributions, and rewards for efficient payment terms.

#### Design

All contracts are accomplished according to best practices through shared BC services.^34^ Examples of various types of contracts include:

(1) British Columbia Renal-driven contracts: PHSA supply chain services is the contracting body, that is, officially organizes the RFPs or invitation to quotes (ITQs), receives bids, organize the scorings, and completes the negotiations following and awarding of the contracts. The day-to-day managing of the contract is a shared responsibility between BCR and PHSA. British Columbia Renal manages operational items such as managing shortages and backorders while PHSA Supply Chain will have any formal communication with the drug company or community pharmacy regarding contract items or supply issues.
(2) Existing group purchasing organization (GPO) contracts such as HealthPro contracts (available to any BC HA): Typically, GPO negotiates for institutional pricing but this may be extended to organizations affiliated with PHSA. British Columbia Renal tends to be the payer of first choice (sole payer) for items purchased through these contracts (due to confidentiality of the pricing). The GN medications and cinacalcet are examples of this arrangement.
(3) British Columbia Renal gets the same pricing as BC PharmaCare Price Listing Agreement (“PLA”) price for some of the products, in keeping with the concept that all funding is ultimately from Ministry of Health.

### Communication and Education

#### Rationale

The rationale is to promote health literacy for patients and enhance the levels of knowledge and competency of health care teams, for the ultimate goal of enhancing access to quality kidney care across BC.

#### Design

British Columbia’s pharmacy services program strives to ensure that appropriate education is provided for health care providers and BCR patients. This is internal to kidney staff, including HA-based renal pharmacists and community pharmacists as well as the patients. Therefore, educational modules, patient and provider facing information sheets, and webinars are provided and updated regularly for patients, pharmacists, and care providers.

##### Education for pharmacists

There are training sessions designed and held for community pharmacists and technicians. There is also a formal process of onboarding renal pharmacists so that they can understand the philosophy of BCR, the processes, the policies, and patient-facing information tools they are expected to use. The Renal Pharmacists network exists as a provincial group bringing together both HA-based and community pharmacists.

The content is provided through live presentations, memos (updates in formulary and processes), one-on-one learning from regional renal pharmacists, zoom calls/webinars, and answering questions via email and telephone.

##### Patients’ education

British Columbia Renal hosts a series of patient education webinars which are targeted at patients with kidney disease, accessible to patients and families. These informative webinars are presented by kidney care providers who work in BC’s kidney programs, as well as by patients with lived experience. Topics range from medications used in CKD, physical activity, mindfulness with kidney disease, understanding laboratory work to kidney transplantation. Presentations and recorded sessions are posted on the BCR website.^35^ Between December 31, 2017 and December 31, 2022, BCR’s patient education website received 7872 page views and 6257 unique page views. However, this is likely an underestimate of the activity as the figures only reflect clicks made on/within the BCR website, and sharing of information downloaded cannot be tracked.

In addition to directed patient education webinars and information sessions, BC’s pharmacy program services provide written handouts for information sheets directed to patients, educating them on the medications that they are receiving. Those information sheets, professionally developed and vetted by patients for readability and understanding, provide knowledge on the drug’s function, mechanism of action, possible side effects, interactions, timing in relation to diet, how to take the medication or what to do in an event of missing a dose. All materials are translated into the common languages spoken by CKD patients in BC: Mandarin, Punjabi, Tagalog, and English. These information sheets can be accessed through a hyperlinks on the BCR’s formulary page on its website.^18-21^ Moreover, a comprehensive series of symptom assessment and management guidelines^36^ together with nonprescription medication guides^37^ developed by renal pharmacists are available to the patients.

### Information Management System

#### Rationale

Information management systems within health care settings are essential to facilitate more accurate clinical decisions, tap into collaborative data sharing, and foster innovation. In fact, data analytics, will help detect hidden patterns in information, delivering actionable insights and enabling systems to predict, infer, and conceive alternatives that might not otherwise be feasible. Such insights help health care systems to improve quality of care, assess cost-effectiveness, identify at-risk populations, and better plan and understand performance.

Taken together, the rationale for incorporating information management system as one of the core components of this pharmacy services system was to enable an integrated provincial system that provides timely, accurate information and innovative analysis to inform the actions and decisions of the health care providers, patients, and policy-makers.

#### Design

Using the province-wide integrated registry and clinical information system, Patient Records and Outcome Management Information System (PROMIS), enables the implementation and evaluation of pharmacy services. This customized information system with provincial interfaces to all laboratories allows for real-time assessment of clinical care, administrative, quality assurance (QA), quality improvement (QI) initiatives and is supported by a group of methodologists and analysts all as part of the BCR. Patient Records and Outcome Management Information System supports all aspects of kidney care planning and delivery and provides real-time, accurate data to support a broad range of functions. It enables health care providers to get access to all aspects of information regarding a patients’ medical care. Data are collected from the 41 renal units and 33 pharmacies in BC in support of individual patient care management, renal unit management, continuous QI, research, and outcome-based planning.

Included in the PROMIS database is tracking of individual patients’ pharmacotherapies. This comprehensive data capture, at a granular individual level, allows assessment of individual prescriptions, assessment of adherence to standardized protocols, and system and outcome assessments. Given that patient outcomes are linked to treatment strategies, PROMIS can be used to assess real-world efficacy of specific medication classes, or combinations; as well as medication utilization. Patient Records and Outcome Management Information System serves both individual patient needs (with customized My Medication Lists), as well as administrative and research purposes. It also includes a medication reconciliation module that provides practice tools (hospital admission orders, hospital discharge orders, medication reconciliation /review, and clinic medication orders) that facilitate seamless transitions in care. Given the integrated and comprehensive data contained within PROMIS, justification for specific medication funding, as well as forecasting use of new medications according to specific conditions (e.g., hyperparathyroidism and hyperkalemia) is possible. Embedded in the system is the capacity to enable automatic approvals (according to predetermined criteria) and evaluating restricted use medications.

## Provincial Pharmacy Services: Mapping to Wagner’s Chronic Care Model

**Figure 2.**
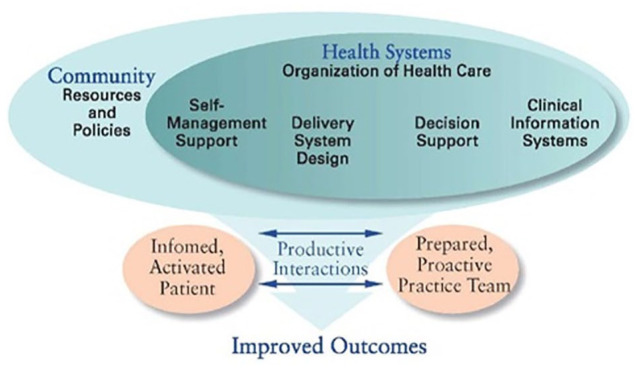
Elements of Wagner’s chronic care model (Wagner, 1999).

Several organizational models for chronic noncommunicable diseases management have been suggested and implemented globally.^38-40^ However, the best known and the most influential model is the Wagner’s CCM which was developed in the 1990s to provide a framework for improvement of quality of chronic disease care.^41-44^ A number of countries have implemented Wagner’s CCM and reported positive outcome measures following interventions.^38,40^ In fact, implementation of Wagner’s CCM model in health care systems is a comprehensive and promising way to operationalize a framework for better care of patients with chronic diseases.^43,45^ The 6 elements of Wagner’s CCM which operate within the context of the individual, community, provider organization, and the health care system are shown in Figure 2. Taken collectively, Wagner’s CCM elements aim to produce effective interactions between proactive practice health care teams and informed activated patients to get better functional and clinical outcomes.^46^

Table 2 shows how our designed pharmacy services program aligns with Wagner’s CCM.

**Table 2.** Alignment of BC Renal’s Pharmacy and Formulary Services Program With Wagner’s CCM.

BC Renal and provincial pharmacy services	Wagner’s CCM					
	Self-management support	Delivery system design	Decision support	Clinical information system	Organization of healthcare	Community linkages (resources and policies)
**Pharmacy Services and Formulary Committee**	✓	✓	✓	✓	✓	✓
**Community of Dispensing Pharmacies**	✓	✓	✓		✓	✓
**Medication and Pharmacy Services Budget**		✓		✓	✓	✓
**Provincial Contracts**		✓			✓	
**Communication and Education**	✓		✓		✓	
**Information Management System**	✓		✓	✓	✓	

*Note.* CCM: chronic care model.

The principal changes (or positive outcomes) involving kidney care through implementation of the provincial pharmacy services program in the context of Wagner’s CCM elements are listed below.

### Self-management support

Self-management support is defined as the systematic provision of education and supportive interventions by health care providers to increase patients’ knowledge, skills, and confidence in their disease management, including regular progress evaluations, goal setting, and problem-solving support.^47^ It ensures patients’ participation in the health care process though developing patients’ self-regulatory and communication skills. Typical self-care activities in the context of pharmacy and pharmaceutical care include adherence to treatment plans and medications, self-monitoring of symptoms and possible side effects of medications, making appropriate decisions concerning when to seek professional assistance and communicating effectively and productively with health care providers and the broader health system.^48^

This element of Wagner’s CCM is enabled through BCR’s PS&F Committee, Community of Dispensing Pharmacies, Communication and Education, and Information Management System in the following ways:

- Securing financial support for patients’ education.
- Including all clinical encounters in the context of pharmaceutical care with a self-management support component.
- Promoting use of BCR patient education brochures and pamphlets created by the PS&F committee and provided by the health care team.
- Access to customized “My Medication Lists” in PROMIS enabling patients to have an active role in their own disease management.

### Delivery system design

It highlights the importance of multidisciplinary health care providers that cover the entire population, serves as a gateway to the system, and integrates health care across levels to meet most of the population’s health needs.^49^ Patients with more complex conditions and/or health care requirements can benefit from clinical case management services from multidisciplinary care providers, who provide close follow-up and help increase adherence.

Delivery system design as one of the elements of Wagner’s CCM is enabled through BCR’s PS&F Committee, Community of Dispensing Pharmacies, Medication and Pharmacy Services Budget and Provincial Contracts in the following ways:

- Direct (systematic) provision of timely and accurate delivery and distribution of kidney and disease-specific medications for patients with kidney disease across all disease trajectories.
- Provision of medication reconciliation for patients with kidney disease in BC.
- Establishment of the provincial BC ADPKD Network, standardization of assessment, and access to disease specific medication (e.g. Tolvaptan).
- Establishment of the GN registry and formulary in BC.
- Provision of care through HA renal pharmacists and contracted quality community pharmacists network in the team.
- Enlargement of kidney care focus in terms of pharmacotherapies from that of an individual visit to include pre-visit, post-visit, and between-visit efforts to organize information and outreach to patients for services they require.
- Provision of relevant community-based services and care and development of partnerships with organizations that support and meet patients’ needs.

### Decision support

Decision support refers to the dissemination of evidence-based clinical practice guidelines, protocols, and best practices across health settings and levels of care.^38,50^

This element of Wagner’s CCM is enabled through BCR’s PS&F Committee, Community of Dispensing Pharmacies, Communication and Education, and Information Management System in the following ways:

- Embedding evidence-based guidelines into daily clinical practice and pharmaceutical care by dissemination of pharmaceutical care guidelines through all contracted pharmacies and renal facilities.
- Promoting clinical and pharmaceutical care that is consistent with scientific evidence.
- Provision of specialist support (through HA renal pharmacists and contracted quality community pharmacists network) to patients.
- Sharing evidence-based guidelines and information with patients to encourage their participation.
- Assessing patient outcomes with regards to different pharmacotherapies through PROMIS.
- Predicting use of new medications in terms of outcomes, safety, and cost-effectiveness through PROMIS.

### Clinical information system

Clinical information systems organize and categorize clinical data on individual patients and the entire clinical populations to facilitate efficient and effective care through assessing health outcomes, monitoring responses to treatment, facilitating identification of patients’ needs, and planning health care over time.^38^

This element is adopted through PS&F Committee, Medication, and Pharmacy Services Budget, and Information Management System in the following manner:

- Implementation of a comprehensive electronic medical records at granular individual level, allowing assessment of individual prescriptions, generation of prescriptions to be filled at a community pharmacy or hospital pharmacy, medication reconciliation, assessment of adherence to standardized protocols, evaluation of clinical outcomes, and the entire system.
- Establishment of GN registry and formulary in BC as an initiative.
- Establishment of provincial BC ADPKD Network, standardization of assessment, and access to medication.
- Track clinical measures and identifying patients who needed education or increased case management.

### Organization of health care

Organization of health care creates a culture, organization, and mechanisms that promote high-quality, safe, and sustainable care. This element clearly paves the way for improvement at all organization levels and promotes effective improvement strategies aimed at comprehensive system change. It also promotes the development of agreements that facilitate care coordination within and across organizations and encourages open and systematic handling of errors and quality problems to improve care.^38^

This element of Wagner’s CCM is adopted through BCR as a provincial organization: attempting to organize the totality of health care for CKD populations in an integrated manner. With respect to pharmacy services specifically, each of the components—PS&F Committee, Community of Dispensing Pharmacies, Medication and Pharmacy Services Budget, Provincial Contracts, Communication and Education, and Information Management System—helps to enable a responsive organization in the following ways:

- Creating a culture, organization, and mechanisms that promote high-quality, safe, cost-effective, and sustainable kidney care.
- Active organizational leadership actions to enable program planning and encouraging and supporting the spread and standardization of best practices by contracting quality pharmacies for delivery of disease-specific medication and pharmaceutical care services to patients with kidney disease.
- Development of partnerships with organizations that support and meet kidney patients’ needs.

### Community linkages (resources and policies)

Health systems that establish formal linkages with their communities leverage untapped resources and provide healthy and facilitative environments for people living with chronic diseases.

Community linkages through recourses and policies are enabled though BCR’s PS&F Committee, Community of Dispensing Pharmacies, and Medication and Pharmacy Services Budget in the following ways:

- Mobilization of community resources to meet unique patients’ needs and encourage patients to participate in effective community programs.
- Provision of patients’ surveys and tools to gain their feedback on equitable access to medication and quality health care.
- Establishment of the health-related community initiatives such as provincial BC ADPKD Network.
- Establishment of the health-related community initiatives such as GN network in BC.

## Limitations

Limitations of the current report include lack of formal evaluation of the program on patient outcomes, but this is relative as the intention of this article is to describe the program which has existed for over 20 years, and is fully functional. Formal evaluation of a complex system would include by costs, cost avoidance, provider, and patients’ satisfaction. We are developing a formal plan for future publications. Detailed analysis and description of the “indirect” costs of establishing and sustaining such a system is important but beyond the scope of this report.

## Conclusion

The BCR pharmacy services program is a provincial pharmacy services, medication access, and distribution system embedded within a provincial network delivering care and services to patients with kidney disease throughout the spectrum of disease. The program has been created to remove barriers to adherence (cost, access, and education), enhance adherence to evidence-based care (protocols, education, evaluation) while ensuring good stewardship of publicly funded health care dollars. Within the context of the BCR budget, pharmacy services are managed in a holistic manner. This is unlike most conventional institutional pharmacies, or Pharmacy and Therapeutics committees, which negotiate the best contract for the institutions or for the drug itself. British Columbia Renal’s pharmacy services program negotiates the best contract for the patient population in a closed system, enables the implementation of province wide value propositions, reviews data related to usage and budget constraints, and is responsible for educational material, and dissemination activities. There are a range of pharmacy services that the provincial system provides beyond the responsibility for budget of the drug formulary. This mature system which has developed and evolved over time consists of the 6 components described above, which map to Wagner’s CCM elements. Successful implementation of the CCM requires supportive policies and financing mechanisms. The model should be considered in its entirety because all its components have synergistic effects, where the whole is greater than the sum of the parts. This provincial system allows for universal coverage and care that is fully funded at the point of service and is described here as an exemplar to develop health systems and services and promote models of care which advance universal access and potentially facilitate the adoption of similar systems in other jurisdictions.
